# Review on treatment of pleural metastasis and malignant pleural effusion with Pressurized IntraThoracic Aerosol Chemotherapy (PITAC)

**DOI:** 10.1515/pp-2023-0048

**Published:** 2024-05-17

**Authors:** Pernille Schjødt Hansen, Martin Graversen, Sönke Detlefsen, Michael Bau Mortensen

**Affiliations:** Odense PIPAC Center (OPC) and Odense Pancreas Center (OPAC), Odense University Hospital, Odense, Denmark; Department of Surgery, HPB and Upper GI Section, Odense University Hospital, Odense, Denmark; Institute of Clinical Research, Faculty of Health Sciences, University of Southern Denmark, Odense, Denmark; Department of Pathology, Odense University Hospital, Odense, Denmark

**Keywords:** cytoreductive surgery, malignant pleural effusion, malignant pleural mesothelioma, palliative treatment, pressurized intrathoracic aerosol chemotherapy

## Abstract

**Background:**

Malignant pleural effusion (MPE) is a common and debilitating condition seen in advanced cancer disease, and life-expectancy is short. Symptoms include pain and severe shortness of breath. Current first-line treatment options include pleural drainage using catheters as well as pleurodesis. However, these treatment modalities are often inefficient and patients need repeated procedures. Pressurized IntraThoracic Aerosol Chemotherapy (PITAC) is a minimally invasive procedure, where antineoplastic agents are nebulized under pressure into the pleural space.

**Content:**

We present the preliminary safety, feasibility, and response assessment data for PITAC based on a comprehensive literature review.

**Summary:**

Five retrospective studies reported data on 38 PITACs in 21 patients. Data were heterogeneous and incomplete on several important aspects such as procedure, safety, local effect and long-term outcomes. PITAC seems technically feasible with a low risk of complications and may provide some reduction in MPE in selected cases.

**Outlook:**

PITAC seems feasible, but prospective phase I and II studies are needed to define safety, indications, and efficacy.

## Introduction

Malignant pleural effusion (MPE) is caused by the spread of malignant cells to the pleural cavity, with increase in fluid production and reduced fluid absorption [1], [2], [3]. It occurs most frequently in malignant mesothelioma, lymphoma or lung, breast, ovarian, and gastrointestinal (GI) cancers, and may itself promote tumor growth and chemotherapy resistance [4], [5], [6], [7]. Presence of MPE is related to a reduced life-expectancy, ranging from three to 12 months depending on primary tumor [2, 5]. Patients present with progressive shortness of breath, weight loss, cough, chest pain, and a reduced quality of life [1, 7, 8]. MPE is found in 15 % of all cancer patients, and accounts for 20 % of the total number of registered patients with pleural effusions in Denmark [8], [9], [10].

Current treatment options include ultrasound-guided pleurocentesis, indwelling pleural catheters (IPC), and chemical pleurodesis [3, 8]. Complications related to these procedures include chest pain, pneumonia or empyema, pneumothorax, catheter related infections, and tract seeding of malignant cells [3, 5, 8, 11]. Hyperthermic intrathoracic chemotherapy (HITHOC) with or without cytoreductive surgery (CRS) has also been used to treat MPE [12, 13]. It can increase tumor cell death, as heat increases the permeability, cytotoxicity, and cytotoxic drug penetration [13]. Still, a systematic review of HITHOC for MPE showed that symptomatic control was achieved in 59–88 % of the patients which was inferior to other forms of surgical pleurodesis [12]. Surgical procedures, such as pleurectomy and pleuro-peritoneal shunting, have been abandoned due to high complication rates and morbidity [3]. In general, the available treatments focus on symptomatic relief, but with suboptimal results, since more than 90 % of the patients require repeated interventions and hospitalization [3, 10, 14].

The lack of effective treatments for recurrent MPE is a significant clinical problem – especially in long term survivors who experience drawbacks of repeated pleurocentesis or complications to IPC and pleurodesis. The ideal treatment of MPE must provide a substantial reduction in the need for fluid evacuation based on a limited number of minimal invasive interventions, but this has not been possible so far.

### Pressurized IntraThoracic Aerosol Chemotherapy (PITAC)

Pressurized IntraThoracic Aerosol Chemotherapy (PITAC) is a minimally invasive platform for repetitive nebulization of antineoplastic agents to the pleural cavity [15]. It is based on the technology and experience from Pressurized IntraPeritoneal Aerosol Chemotherapy (PIPAC), which is increasingly used to treat patients with peritoneal metastasis (PM) from different primary tumors. Preclinical data show a more homogenous distribution as well as a deeper penetration of anti-neoplastic agents in PIPAC when compared to lavage-based intraperitoneal chemotherapy [4, 15, 16]. Clinical data point towards a local effect on PM according to the Peritoneal Regression Grading Score (PRGS), and also a potential reduction in ascites [17], [18], [19], [20], [21].

The first PITAC procedure was performed in 2012, but the procedure has not been widely implemented, and there is no consensus on indications, techniques, or methods of response evaluation. This is a comprehensive review of the published literature on patients treated with PITAC.

## Materials and methods

A literature search was conducted in PubMed ending October 31^st^, 2023. Publications in English that described PITAC directed therapy in humans with MPE and/or pleural metastasis (PLM) were included. No publication status was imposed. The studies were assessed by manual review of the abstracts followed by a full-text review. A backward citation search was done on included studies to ensure inclusion of all relevant studies. Additionally, ClinicalTrials.gov (October 31^st^, 2023) was screened for ongoing or completed PITAC trials [22].

To assess feasibility, patient safety, and response to PITAC directed therapy, the following outcomes were reviewed: patient characteristics, indications, prior cancer directed therapy, technical aspects of the PITAC procedure including occupational health, pleural biopsies, pleural cytology, intra- and postoperative complications, patient reported outcomes (PRO), length of stay (LOS), mortality, and number of PITACs.

## Results

The literature search yielded 15 potential articles. After review of the abstracts, 12 of these did not describe data on PITAC directed therapy. The remaining three studies were included [23], [24], [25], and a backward citation search led to the inclusion of two additional studies [4, 26]. The included studies were retrospective case series with 10 or less patients. No planned or ongoing PITAC trials were found on ClinicalTrials.gov [22].

A total of 38 PITAC directed therapies were performed in 21 patients with MPE and/or PLM of which 10 patients received two or more PITACs (Table 1).

**Table 1:** Number of included patients and PITAC procedures.

	Patients n (%)	1 PITAC n (%)	2≥PITAC n (%)	Total PITAC procedures n
Jonscher et al. (2012–2014) [4]	6 (100)	5 (83.3)	1 (16.6)	10
Giger-Pabst et al. (2014) [25]	3 (100)	1 (33.3)	2 (66.6)	5
Kuchen et al. (2014–2018) [26]	10 (100)	3 (30)	7 (70)	21
Robella et al. (2018) [24]	1 (100)	1 (100)	0 (0)	1
Drevet et al. (2020) [23]	1 (100)	1 (100)	0 (0)	1
Total	21	11	10	38

PITAC, Pressurized IntraThoracic Aerosol Chemotherapy; n, number; %, percentage.

### Patient characteristics

Two studies evaluated both PITAC and PIPAC directed therapy, which made it impossible to extract specific characteristics regarding PITAC patients [25, 26]. Another study did not provide information on patient characteristics including prior treatments [4]. The remaining studies included patients with gastric or ovarian cancer, pseudomyxoma peritoneii, or malignant mesothelioma [23, 24]. Indications for PITAC directed therapy were MPE, PLM or microscopic residual disease (Table 2).

**Table 2:** Patient characteristics.

	Jonscher et al. [4]	Giger-Pabst et al. [25]	Kuchen et al. [26]	Robella et al. [24]	Drevet et al. [23]
Gender (F/M)	N/A	N/A^a^	N/A^a^	0/1	0/1
Median age, years (range)	N/A	N/A^a^	N/A^a^	50	72
Median ECOG performance status (range)	N/A	N/A	N/A^a^	N/A	N/A
Cancer origin, n	Gastric (n=4) Ovarian (n=1) Malignant mesothelioma (n=1)	Malignant mesothelioma (n=3)	N/A^a^	Pseudomyxoma peritoneii	Gastric
Prior therapies	N/A	N/A^a^	N/A^a, b^	N/A^c^	Bidirectional^d^
Indication for PITAC	MPE	MPE	PLM	Microscopic residual disease	MPE

^a^PITAC and PIPAC data merged. ^b^Inclusion criteria was previous non-successful chemotherapy. ^c^Peritoneal PMP treated with chemotherapy and surgery, but no information on prior treatments for MPE. ^d^Chemotherapy and PITAC. ECOG, Eastern Cooperative Oncology Group; PMP, pseudomyxoma peritonei; PLM, pleural metastasis; MPE, malignant pleural effusion.

### PITAC procedure

The PITAC procedures were performed in general anesthesia using a double lumen endotracheal tube to allow exclusion of the ipsilateral lung [4, 23], [24], [25]. In two studies the patients were in lateral decubitus position [23, 25], whereas the remaining three studies did not describe the positioning of the patients (Table 3) [4, 24, 26]. When reported, pleural access was obtained along the anterior axillary line (AAL) and the midaxillary line (MAL) at the fifth to eighth intercostal space (IC) using 5- and 12 mm balloon trocars (Table 3) [4, 23], [24], [25], [26].

**Table 3:** Reported techniques, intraoperative assessment, complications and follow-up after PITAC directed therapy.

	Jonscher et al. [4]	Giger-Pabst et al. [25]	Kuchen et al. [26]	Robella et al. [24]	Drevet et al. [23]
Positioning	N/A	Lateral (n=3)	N/A	N/A	Lateral
Balloon trocar placement	N/A	IC 6–8	N/A	IC 7 IC 8	MAL IC 7 AAL IC 5
Balloon trocar size	N/A	12 and 5 mm	N/A	N/A	12 mm
Assess pleural involvement	N/A	N/A	Yes^a^	Yes^b^	Yes
Mean volume of MPE (range)	N/A	First PITAC: 1150 mL (900–1900) Follow-up: 250 mL (200–300)	First PITAC: 900 mL (0–1800) Last PITAC: 450 mL (0–900)	N/A	N/A
Cytology of MPE	N/A	N/A	N/A	N/A	N/A^c^
Biopsies taken	Yes	Yes^d^	N/A	No^b^	Yes
Pleural lavage	N/A	N/A	N/A	N/A	N/A
Safety checklist	N/A	N/A	Yes	N/A	Yes
Applied chemotherapy (mg/m^2^)	Cisplatin 7.5 and doxorubicin 1.5	Cisplatin 7.5 and doxorubicin 1.5	N/A^d^	Cisplatin 7.5 and doxorubicin 1.5	Cisplatin 10.5 and doxorubicin 2.1
Diffusion time, min	30	N/A	30	30	30
Synchronous surgeries	PIPAC^e^ (n=1)	PIPAC (n=3)	Wedge resection (n=2)	Resection of 7^th^ rib Complete parietal pleurectomy Partial visceral pleurectomy Small lung resections (n=3)	No
Chest tube	N/A	Yes^f^	N/A	Yes	Yes
Postoperative routine X-ray	N/A	Yes	N/A	Yes	N/A
Prophylactic antibiotics	N/A	N/A	N/A	N/A	N/A
Intraoperative complications	No	No	No^g^	N/A	No
Postoperative adverse events	CTCAE<2: N/A CTCAE>2: No	N/A^dh^	Yes^dg^ (n=2)	No	No
Length of stay, days	N/A	N/A	N/A^d^	11	N/A
Mortality	0	N/A	N/A	N/A	N/A
MPE control^I^	Yes (n=6)	Yes (n=2)	Yes (n=3)	Yes	Yes
Follow-up procedure(s)	N/A	CT-scans	N/A	N/A	Clinical assessment and X-ray

^a^Extent of pleural carcinomatosis (EPC). ^b^Pleurectomy and lobectomies prior to PITAC therapy. ^c^Indication were positive cytology. ^d^PITAC and PIPAC data merged. ^e^One patient received four simultaneous PIPACs. ^f^Removed immediately after successful re-ventilation. ^g^Clavien-Dindo classification. ^h^CTCAE classification. ^I^Reported as positive response or stabile volume. IC, intercostal space; AAL, anterior axillary line; MAL, midaxillary line; CTCAE, Common Terminology Criteria for Adverse Events; MPE, malignant pleural effusion; CT, computerized tomography.

An intrathoracic pressure of 12 mmHg at 37 degrees Celsius was maintained throughout the procedures, and a CE-certified nebulizer was inserted through the 12 mm trocar [4, 26]. Two studies did not report whether normo-thermic carbon dioxide was applied or not [23, 25], and one study did not report whether intrathoracic pressure was maintained [24]. The use of a specific safety checklist was reported in two studies [23, 26]. The staff left the operating room before initiating PITAC by remote control in three studies [4, 23, 24]. Standard doses used for PIPAC were also used for the PITAC procedures (Oxaliplatin 92 mg/m^2^, cisplatin 7.5 or 10.5 mg/m^2^ and doxorubicin 1.5 or 2.1 mg/m^2^) [4, 23], [24], [25], [26]. After a diffusion time of 30 min, the aerosol was evacuated through a closed ventilation system [4, 23], [24], [25], [26].

Visual assessment of PLM was performed in three studies [23, 24, 26], and MPE volume was quantified and analyzed in two studies [25, 26]. None of the studies performed pleural lavage if no or only small amounts of MPE were present. The number and type of previous interventions to relieve symptoms caused by MPE were not reported in any of the studies [4, 23], [24], [25], [26].

Biopsies for histology verification and response evaluation were obtained in three studies [4, 23, 25]. Pleurectomy and several lobectomies were performed in one study before PITAC, but no additional biopsies were taken [24]. Although not specifically stated, the last study also seemed to have taken biopsies for response evaluation [26].

Placement and size of chest tubes after PITAC also varied. Giger-Pabst et al. placed a Charriere 12 chest tube in a ventro-apical position connected to a digital drainage system with a constant negative pressure of 15 mmH_2_O. After re-ventilation of the ipsilateral lung, the chest tube was removed [25]. Drevet et al. placed a Charriere 24 chest tube in a postero-apical position with a constant negative pressure of 20 mmH_2_O, while Robella et al. placed two chest tubes through the trocar access points (Table 3) [23, 24]. Jonscher and Kuchen et al. did not describe the re-ventilation process [4, 26], and no information on tube removal was available in general [4, 23], [24], [25], [26]. Postoperative chest X-rays were used in two studies [24, 25]. The use of prophylactic antibiotics was not disclosed in any of the studies. No intraoperative events that may have posed a potential risk to the occupational health safety were reported [4, 23], [24], [25], [26].

### Intra- and postoperative complications

Two studies used either Common Terminology Criteria for Adverse Events (CTCAE) or Clavien-Dindo to classify postoperative complications [25, 26]. Neither intraoperative complications [4, 23], [24], [25], [26] nor any CTCAE>2 were reported, but one study reported a mixture of postoperative complications/events related to PIPAC and PITAC. Thus, isolated data on PITAC were not available [25]. Kuchen et al. reported two Clavien-Dindo grade I incidences of prolonged air-leakage after simultaneous lung wedge resections, which they treated conservatively. These two complications were probably not related to the PITAC procedure [26]. Robella et al. stated that the postoperative course was uneventful, and no cardiovascular or pulmonary complications were observed. Drevet et al. also stated that the postoperative course was uneventful, but none of the two studies used any classification system [23, 24].

### Postoperative outcomes

#### Control of MPE

Jonscher et al. detected no significant MPE for the included patients at two-month follow-up [4]. Giger-Pabst et al. had one patient with MPE relapse four weeks after the first PITAC therapy. Six months after the first PITAC, the last two patients had unchanged MPE of 200 and 300 mL, respectively [25]. Kuchen et al. reported that three out of seven patients had a positive effect between the first and second PITAC [26]. Six months after PITAC directed therapy, Robella et al. found no recurrence of MPE and Drevet et al. made the same observation three months after PITAC [23, 24].

#### Biopsies and cytology

Jonscher et al. reported one patient with malignant mesothelioma that had an additional PITAC procedure on the same side, and one of multiple biopsies taken at PITAC2 showed a major pathological response to PITAC [4]. In another study, biopsies were obtained to enable the histological response assessment in the event that more PITAC procedures were performed. Unfortunately, since only one PITAC procedure was performed, the response could not be assessed [23]. Giger-Pabst et al. assessed the histological tumor response by tumor regression grade (TRG) as described by Dworak [27], but the response data were merged with PIPAC data hindering PITAC induced response evaluation [25]. One study did not publish information on biopsies taken in relation to the PITAC procedures apart from the Ki-67 proliferation index, and it was not specified how this variable was determined [26].

None of the published studies used cytology for response evaluation.

#### PRO data

No PRO data (e.g., quality of life) specifically related to the PITAC procedure were reported.

#### Length of stay

The LOS was only reported in one study of a patient undergoing synchronous major resection [24].

#### Mortality

No studies reported mortality in relation to PITAC directed therapy.

### Follow-up

Giger-Pabst et al. performed CT-scans six months postoperatively [25]. Drevet et al. performed monthly x-rays and clinical assessment three-months postoperative [23]. Three studies provided no data on follow up procedures [4, 24, 25].

## Discussion

Treatment of MPE (and PLM) is difficult for several reasons, and no ideal treatment strategy is available. The optimal treatment should relieve symptoms, be minimal invasive, have a low risk of complications, a minimum LOS, and (re-)interventions [3]. Based on the experience from PIPAC directed therapy in patients with PM, PITAC has been suggested for use in patients with MPE and/or PLM.

This review identified five minor case series on PITAC directed therapy with a total of 38 PITAC procedures in 21 patients [4, 23], [24], [25], [26]. The studies were retrospective and heterogenous in reporting details related to both patient characteristics, indications, procedures, treatment response, and outcomes. Some studies merged data from both PIPAC and PITAC directed therapy, making it difficult to specifically evaluate the PITAC procedure and related outcomes.

Primary tumors included malignant mesothelioma, gastric- and ovarian cancer, and pseudomyxoma peritoneii. One study performed PITAC solely on PLM, three studies solely on clinically relevant MPE and one study to eliminate microscopic residual disease. It is difficult to evaluate whether PITAC directed therapy could be a treatment option for both PLM and MPE. Repeated PITAC procedures conveyed a positive response in some patients, such as reduced MPE, but the local efficacy of PITAC remains uncertain. No data evaluating the potential clinical and prognostic impact of PITAC induced conversion from malignant to benign pleural cytology were available.

Due to the many limitations of the presented data, it is impossible to estimate whether PITAC may be an alternative to the traditional treatment of MPE. The rate of complications from repeated ultrasound-guided pleurocentesis depends on the number of performed procedures, as the risk accumulates over time due to increased inflammation and loculation [3]. Similar, chemical pleurodesis with talcum causes a severe inflammatory reaction with cough, pain, and fever [3]. Due to the limited PITAC follow up data, similar negative long-term outcomes (e.g., chronic inflammation, loculation, fistulas, etc.) were impossible to evaluate.

Another significant difference between some of the current treatment options and PITAC is the need for general anesthesia. This may entail an increased risk for patients already respiratory affected. Furthermore, the application of a double-lumen endotracheal tube – when or if necessary, requires specialized anesthesiologists.

Despite the focus on occupational health safety issues in relation to PIPAC directed therapy, such data were lacking in the present PITAC studies [28, 29]. The procedure was performed with different setups and in some centers without a dedicated PITAC safety checklist [4, 24, 25]. Such a checklist should be based on the experience from future phase I studies, and should be linked to a standard operating procedure for PITAC – including suggestions for postoperative observation and monitoring [30].

This review is limited by the inclusion of only five small retrospective case series. Data on patient selection, previous treatment, indications, technical aspects of the PITAC procedure, intra- and postoperative complications, response assessments, clinically relevant outcomes, and follow-up are poorly defined and data from serial procedures are lacking. To evaluate the treatment effect of PITAC on either MPE or PLM, future studies may need to assess each entity separately. However, since patients can have MPE and PLM synchronously, treatment response may be investigated by monitoring both fluid reduction, histology, and cytology.

Finally, since patients with MPE and PLM have a poor prognosis, treatment evaluation must include patient reported outcomes to investigate whether an invasive procedure like PITAC is justified in these patients.

When a novel surgical intervention is to be implemented and standardized, the IDEAL framework provides recommendations regarding design, development, and reporting. The implementation should be done in the following stages – idea, development, exploration, assessment, and long-term studies [16]. The included studies suggest that the PITAC procedure is still at the idea stage (phase 1) and possibly moving to the development stage (phase 2a). Some of the presented studies have concluded that PITAC directed therapy is safe, however, well designed and larger prospective phase I trials are needed before any firm safety conclusions can be made, and before moving on to the next level of evidence.

## Conclusions

PITAC is on the verge of entering phase 2a of the IDEAL framework, but standard operating procedures, response assessment, safety issues and monitoring strategies are lacking. Prospective data focusing on feasibility, safety, and efficacy are needed, and this will require strict definitions regarding patient selection, PITAC procedure, perioperative patient handling, response evaluation, and outcome assessment.
